# Glycine betaine biosynthesis genes differentially expressed in sugarcane under water stressGlycine betaine biosynthesis genes differentially expressed in sugarcane under water stress

**DOI:** 10.1186/1753-6561-8-S4-P123

**Published:** 2014-10-01

**Authors:** Sonia Zingaretti, Paula Demore, Thaiza Morceli, Luana Mantovanini

**Affiliations:** 1 Universidade de Ribeirão Preto - UNAERP, Ribeirão Preto, Brazil; 2 Universidade Estadual Paulista - UNESP-Jabuticabal, Jabuticabal, Brazil

## Background

Plants have developed a wide range of strategies which allow their survival under abiotic stresses [1]. One of those mechanisms is the accumulation of compatible solutes, which protect cell structure against damage induced by dehydration and oxidation [2]. The accumulated compatible solutes may include betaines and related compounds such as sugars and amino acids. Understanding the action mechanism of glycine betaine (GB) and its effects on drought tolerance mechanisms may lead to the development of cultivars adapted to different hydric conditions. In medicinal plants, betaine is synthetized by two enzymes: choline monooxygenase (CMO), and betaine aldehyde dehydrogenase (BADH) [3]. The present study evaluated gene expression profiles of CMO and BADH genes in sugarcane leaves (Saccharum SP) submitted to three levels of water stress (mild, moderate and severe), using DNA microarrays.

## Methods

Sugarcane plantlets of cultivars SP83-2847 and SP90-1638, considered moderately tolerant and sensitive to water stress, respectively, were cultivated in greenhouse for 60 days in sterilized soil at 26ºC with 56% of humidity. After 2 months, water stress was induced by irrigation suppression. All leaves were collected when water content in the soil was 10%, 50% and 75% respectively, and designated as mild, moderate and severe water stress.

Total RNA was extracted using Trizol Reagent (Invitrogen, USA), according to the manufacturer's instructions and used to synthesize a cDNA probe which was used to hybridize nylon membranes containing CMO and BADH genes. Membranes were hybridized, for 18h at 58°C, probe was washed out and filters were exposed to imaging plates (Fujifilm, Japan) for 96 hours. Images were scanned using Phosphorimager FLA3000-G (Fujifilm, Japan), and spots quantified by Array Vision (Imaging Research, Canada). Variation in gene expression levels was obtained by log2 of the ratio of values found for control plants and plants under water stress during different trial periods. Northern Blot was used for validation of the results obtained by the microarray.

## Results and conclusions

Our analyses showed an increase in the gene expression level for BADH enzyme only under moderate and severe stress for the sensitive cultivar, where differential expression of the tolerant variety started at the beginning stress. No differential expression was observed for CMO at significant levels for both varieties. These results confirm BADH gene modulation under water stress and its relationship with water stress tolerance.
